# Effects of vitamin D supplementation in extender on sperm kinematics and apoptosis following the freeze-thaw process in normozoospermic and asthenozoospermic Holstein bulls

**DOI:** 10.1186/s12610-021-00137-5

**Published:** 2021-08-05

**Authors:** Reza Asadpour, Morteza Taravat, Maryam Rahbar, Mohammadrasoul Khoshniyat, Gholamreza Hamidian

**Affiliations:** 1 Department of Clinical Science, Faculty of Veterinary Medicine, University of Tabriz, Tabriz, Iran; 2 Research Center of Iranian Nahadehaye Dami Jahed (NDJ), Karaj, Iran; 3 Department of Basic Science, Faculty of Veterinary Medicine, University of Tabriz, Tabriz, Iran

**Keywords:** Vitamin D, Sperm kinematics, Apoptosis, Normozoospermic, Asthenozoospermic, Vitamine D, Cinématique des Spermatozoïdes, Apoptose, Normozoospermie, Asthénozoospermie

## Abstract

**Background:**

Asthenozoospermia is a usual male infertility factor, characterized by decreased semen quality. It has been revealed that antioxidants improve sperm function, enhance endogenous antioxidant activities, and protect spermatozoa against oxidative damage during cryopreservation. This aimed to evaluate the effects of vitamin D on sperm kinematics and apoptosis in the semen of bulls with normozoospermia and asthenozoospermia after the freeze-thaw process. For this purpose, 32 semen samples of four Holstein bulls (normozoospermic, progressive motility > 70 %) and 32 semen samples of four bull (asthenozoospermic progressive motility < 40 %) were collected and pooled separately (normozoospermic and asthenozoospermic). Samples were then diluted into four equal aliquots of extender containing different vitamin D concentrations (0, 5, 10, and 50 ng/mL) and aspirated into a 0.5 mL straw.

**Results:**

The percentages of sperm progressive motility and viability were significantly higher (*P* < 0.05) in 50 ng/mL of vitamin D in normozoospermic group. Sperm kinematics parameters including curvilinear velocity (VCL), straight-line velocity (VSL), and average path velocity (VAP) were significantly higher in the high dose (50 ng/mL) vitamin D-treated group compared to the low dose vitamin D-treated group (5ng/mL) in normozoospermic bull semen samples. The supplementation of the semen extender with different concentrations of vitamin D could not increase the rate of acrosome integrity in normozoospermic bulls compared to the control group (*P* < 0.05). In the asthenozoospermic group, 10 ng/mL vitamin D-treated group could increase the rate of plasma membrane integrity compared to 5 ng/mL vitamin D-treated group (*P* < 0.05). The percentages of early-apoptosis (*P* = 0.049) and late-apoptosis (*P* = 0.005) were significantly higher in the asthenozoospermic than the normozoospermic group.

**Conclusions:**

The present study revealed that a high dose (50 ng/mL) of vitamin D protected normozoospermic bulls’ sperms from the freezing procedure and lead to higher quality of frozen-thawed bull sperm.

## Résumé

**Contexte:**

L’asthénozoospermie est un facteur courant d’infertilité masculine, caractérisé par une diminution de la qualité du sperme. Il a été montré que les anti-oxydants amélioraient la fonction des spermatozoïdes, augmentaient les activités anti-oxydantes endogènes, et protégeaient les spermatozoïdes contre les dommages oxydatifs lors de la cryoconservation. Cette étude visait à évaluer les effets de la vitamine D sur la cinématique et l’apoptose des spermatozoïdes dans le sperme de taureaux qui présentaient une normozoospermie ou une asthénozoospermie après le processus de congélation-décongélation. À cette fin, 32 échantillons de sperme de quatre taureaux Holstein (normozoospermiques, mobilité progressive >70 %) et 32 échantillons de sperme de quatre taureaux (asthénozoospermiques ; mobilité progressive <40 %) ont été recueillis et regroupés séparément (normozoospermiques et asthénozoospermiques). Les échantillons ont ensuite été dilués en quatre aliquotes égales dans un milieu contenant différentes concentrations de vitamine D (0, 5, 10 et 50 ng/mL), puis aspirés dans une paille de 0.5 mL.

**Résultats:**

Les pourcentages de mobilité progressive et de viabilité des spermatozoïdes étaient significativement plus élevés (*p*<0.05) avec 50 ng/mL de vitamine D dans le groupe normozoospermique. Dans les échantillons de sperme de taureaux normozoospermiques, les paramètres cinématiques des spermatozoïdes, incluant la vitesse curvilinéaire (VCL), la vitesse en ligne droite (VSL), et la vitesse moyenne du trajet (VAP), étaient significativement plus élevés dans le groupe traité par vitamine D à dose élevée (50 ng/mL) que dans le groupe traité par vitamine D à faible dose (5ng/mL). La supplémentation du milieu avec différentes concentrations de vitamine D n’a pas pu augmenter le taux d’intégrité de l’acrosome chez les taureaux normozoospermiques comparés au groupe témoin (*p*<0.05). Dans les échantillons de sperme de taureaux asthénozoospermiques, le groupe traité par vitamine D à la dose de 10 ng/mL a augmenté le taux d’intégrité de la membrane plasmique par comparaison au groupe traité par vitamine D à la dose de 5 ng/mL (*p*<0.05). Les pourcentages d’apoptose précoce (*p*=0.049) et d’apoptose tardive (*p*=0.005) étaient significativement plus élevés dans le groupe asthénozoospermique que le groupe normozoospermique.

**Conclusions:**

La présente étude a montré qu’une dose élevée (50 ng/mL) de vitamine D protégeait les spermatozoïdes des taureaux normozoospermiques lors de la procédure de congélation, et menait à une meilleure qualité des spermatozoïdes congelés-décongelés chez ces taureaux.

## Background

Asthenozoospermia is a usual male infertility factor, characterized by total sperm immobility or very low motile spermatozoa [1]. There are several reasons for asthenozoospermia, such as metabolic and ultrastructural abnormalities, functional deficiencies, genetic defects, physical and chemical factors, anti-sperm antibodies, varicocele, and endocrine abnormality [2].

The freeze-thaw process can reduce sperm motility, viability, and fertilization capacity of spermatozoa [3]. A number of studies have shown that the freeze-thaw process increases sperm DNA fragmentation, apoptosis, and formation of reactive oxygen species (ROS) by non-viable sperm [4]. It has been shown that increased ROS levels after cryopreservation may lead to damaged acrosome integrity, DNA fragmentation, and reduced sperm motility [5], deficiency of sperm morphology, function, and eventually male infertility [6]. This process is mediated by the lipid peroxidation of the sperm plasma membrane, cold shock, osmotic stress, and intracellular ice crystal formation [5]. Several studies revealed that antioxidants improved sperm function, such as sperm motility and integrity, enhanced endogenous antioxidant activities, and protected spermatozoa against oxidative damage [7, 8].

There is evidence from animal studies that vitamin D (Calcitriol) regulates testis function [9, 10]. The vitamin D receptor (VDR) and its metabolizing enzymes are expressed in the germ cells and mature spermatozoa [11]. It has a widespread biological function, including an essential role in the regulation of calcium homeostasis and bone mineralization, transcription of several genes involved in mitotic activities, apoptosis, differentiation of cellular cholesterol homeostasis, stabilization of the chromosomal structure, and regulation of sex steroid hormones [12]. Vitamin D deficiency in rodents reduced sperm counts, impaired sperm motility, and lowered fertility rate in females inseminated with semen from vitamin D-deficient males [13]. Another study showed that vitamin D might positively affect sperm motility [14]. Besides, the supplementation of a boar diet with 2000 IU/kg of vitamin D increased sperm motility [15]. In addition, vitamin D improved sperm viability [16], motility, early apoptosis, and necrotic of sperm were reduced in asthenozoospermic patients [17]. A study on human sperm revealed that adding vitamins to the sperm dilution medium could reduce the rate of apoptosis [17].

In light of the above information, it can be assumed that vitamin D can act as a substance (e.g. an antioxidant) against the harmful effects of the freeze-thaw process in sperm. To this end, a freezing extender was supplemented with vitamin D dose-dependently in semen samples of normozoospermic and asthenozoospermic bulls. After the freeze-thaw process, sperm motility and kinematic parameters were evaluated by computer-assisted sperm analysis (CASA). Furthermore, its effects on the sperm plasma membrane, acrosome integrity, and apoptosis status were examined in both types of bulls.

## Materials and methods

### Animals and semen collection

Eight Holstein bulls (two groups of four bulls with histories of sperm progressive motility < 40 % and > 70 %) were selected and monitored for more than one year and retained with the same feeding management routine at the Iranian Nahadehaye Dami Jahed(NDJ) Company (Karaj, Iran). Semen samples were collected twice a week for 4 weeks using an artificial vagina (AV) according to the standard artificial insemination (AI) technique. Immediately after collection, the samples were kept at 35 °C for further analysis. Shortly after semen collection, sperm progressive motility samples were assessed by a computer-assisted semen analyzer (AndroVision®, minitube). After an initial evaluation, bull semen samples having progressive motility > 70 % and < 40 % were categorized as normozoospermic and asthenozoospermic, respectively. Samples from each group were pooled to minimize the individual variability and attain adequate semen for triplicates. Semen samples having progressive motility above 40 % and below 70 % were excluded from this study.

### Experimental design

In this study, vitamin D3 (Vitamin D) was dissolved in 0.1 % dimethyl sulfoxide (DMSO) to achieve the Vitamin D concentrations of 0, 5, 10, and 50 ng/mL (740,292 Sigma-Aldrich). The selection of Vitamin D concentrations was decided based on a previously published work [18]. The pooled ejaculates in each group were diluted with 250 mmol/L of Tris (Tris-hydroxymethyl amino-methane), 90 mmol/L of citric acid, 70 mmo/L of fructose, 100 IU/mL of penicillin G, 100 µg/mL of streptomycin, glycerol 7 % (v/v), and egg yolk 20 % (v/v) to obtain a final sperm concentration of 25 × 10^6^ spermatozoa/mL per 0.5 ml of straw. The semen samples of each group (normozoospermic and asthenozoospermic) were then divided into four equal aliquots, viz. without vitamin D (control), and those supplemented with 5, 10, and 50 ng/mL of vitamin D, respectively. Extended semen was loaded into 0.5 mL straws (IMV® Technologies, L’Aigle Cedex, France), equilibrated for 4 h at 4 °C, and then frozen using a programmable freezer (Digit Cool®, IMV® Technologies, L’Aigle Cedex, France) (4 to − 10 °C at 5 °C/min; -10 to -100 °C at 40 °C/min, and − 100 to -140 °C at 20 °C/min). Straws were subsequently plunged into liquid N_2_ and stored until assessing after one day. After 24 h, frozen straws were thawed in a water bath at 37 °C for 30 s (*n =* 4 per trial for each concentration) for evaluation tests. Samples were assessed for sperm kinematic parameters, computer-assisted sperm analysis (CASA), plasma membrane integrity, Hypo-Osmotic swelling test (HOST), acrosome integrity, fluorescein-conjugated *Pisum sativum* agglutinin (FITC-PSA), apoptotic statues (Annexin V-FITC), and ROS levels.

### Sperm motion characteristics

For this purpose, 5 µL of semen was placed into a pre-warmed chamber slide (38 °C, 4, 20 μm height; Leja® slide of CASA system, AndroVision®, minitube), which was previously set up to assess the bull sperm [19]. This system was used to evaluate different parameters, such as sperm total motility (TM, %), progressive motility (PM, %), average path velocity (VAP, µm/s), curvilinear velocity (VCL, µm/s), amplitude of lateral head displacement (ALH, µm), beat/cross-frequency (BCF, Hz), straightness (STR, %), linearity (LIN, %), distance straight line (DSL, µm), distance of average path (DAP, µm), straight linear velocity (VSL, µm/s), and wobble (WOB, %). At least 200 spermatozoa were assessed in each CASA analysis [20].

### Sperm viability

The percentage of sperm viability was assessed using eosin-nigrosine staining procedures [21]. For this purpose, approximately equal volumes of semen and stain (5 + 5µL) were mixed and smeared using a second slide. To calculate viable and non-viable spermatozoa, at least 200 cells were counted in at least five different fields of bright microscopy (Scope.A1 ZEISS) at 1000 X magnification.

### Sperm plasma membrane integrity

The evaluate sperm plasma membrane by the hypo-osmotic swelling (HOS) test [22], 30 µL of semen samples were mixed with 300 µL of a hypoosmotic solution (13.5 g of fructose and 7.35 g of sodium citrate dissolved in 1 L water with osmolality of 100 mOsm/kg) and incubated at 37 °C for 60 min. The smear slide was homogenized and evaluated by a phase-contrast microscope (400x magnification). A total of 200 spermatozoa was counted in at least six different fields of the microscope to record the percentage of spermatozoa with curled (as an appositive to test) and non-curling (straight) tails.

### Acrosome integrity

The evaluate acrosome integrity according to Thys et al. [23], 500 µL of the sperm solution was put into a microtube. After centrifugation and removal of the supernatant, the sperm pellet was solved in 100 µl of ethanol (96 %) and kept at room temperature for 30 min. Subsequently, 10 µl of sperm suspension was put on a glass slide, mixed with 30 µl of FITC-PSA, incubated at room temperature for 20 min, and then dripped 10 times in distilled water. They were allowed to air-dried and mounted with glycerol. Totally, 200 sperm per slid were measured by a fluorescence microscope (BX51, Olympus) at 400x magnification. Sperm head with green fluorescent was detected as intact acrosome, and those lacking green authority were considered disrupted or damaged acrosomes.

### Phosphatidylserine (PS) externalization

To recognize the externalization of phosphatidylserine as an indicator of apoptosis, sperm samples were washed in calcium (Ca) buffer and re-adjusted to a concentration of 1 × 10^6^ spermatozoa/mL [24]. Then, 10 µL of Annexin V-FITC was added to 100 µL of sperm solution and incubated on ice for 20 min. Lastly, 10 µL of propidium iodide (PI) was applied to the sperm suspension and incubated on ice for 10 min. For each sample, 10,000 events were counted by flow cytometry (Becton-Dickinson, San Khosoz, CA, USA). Sperm were classified into four group: (1) viable or non-apoptotic cells negative for Annexin-V and exclude PI (Propidium Iodide) staining (A^−^/PI^−^); (2): early apoptotic cells that bind Annexin V but exclude PI (A^+^/PI^−^); (3):late apoptotic cells that bind both Annexin-V and PI (A^+^/PI^+^); and(4): necrotic cells that exclude Annexin-V and bind PI (A^−^/PI^+^). Green fluorescence and propidium (red fluorescence) were measured by 530/30 nm (FL1) and 585/42 nm (FL2) band-pass filters, respectively.

### Evaluation of ROS production

The ROS level was measured using a dichlorofluorescin diacetate probe (DCHF-DA, D6883, Sigma-Aldrich). After the freeze-thaw process, 20 µl of DCFH-DA stain mixed with 100 µl of the semen sample and incubated in the dark at 25 °C (room temperature) for 30 min. DCFH-DA was analyzed using the flow cytometric method [25].

### Statistical analysis

Each experiment was replicated four times. All data were evaluated for normal distribution by the Shapiro-Wilk test. Data were tested for homogeneity of variances using Levene’s test. Then, data of sperm kinematic parameters, apoptosis status, sperm membrane functionality, and ROS production in two groups of bulls were analyzed with nested-ANOVA using SPSS version 26.0. Duncan’s multiple range test distinguished statistical discrepancy among the different groups, and values of *P* ≤ 0.05 were considered statistically significant. Results are expressed as mean ± standard error of mean (SEM).

## Results

### Semen quality and kinematic parameters

The value of sperm kinematics and the percentages of total motility, progressive motility, and viability of sperm are presented in Tables 1 and 2, respectively. Significantly higher rates (*P* < 0.05) of progressive motility and viability were observed in 50 ng/mL of vitamin D in the normozoospermic group. However, the lowest sperm viability and progressive motility were achieved by adding 10 and 50 ng/mL of vitamin D to the asthenozoospermic semen extender compared with the control group. Sperm total motility was affected by none of the vitamin D concentrations in both normozoospermic and asthenozoospermic groups after the freeze-thaw process. Sperm kinematics parameters including VCL, VSL, and VAP were significantly higher in the high dose (50 ng/mL) vitamin D-treated group compared to the low dose vitamin D treated group (5ng/mL) in normozoospermic bull semen samples. However, no remarkable differences were observed in the sperm kinematic parameters of asthenozoospermic samples at all vitamin D concentrations.

**Table 1:** Effect of supplementation of semen extender with different concentration of vitamin D on frozen–thawed spermatozoa kinematics in normozoopsermic and asthenozoopsermic Holstein bulls

Vit D (ng/mL)	Normozoospermic bull				Asthenozoospermic bull			
	**0**	**5**	**10**	**50**	**0**	**5**	**10**	**50**
DCL(µm)*	32.21 ± 1.82	32.21 ± 0.42	30.88 ± 2.04	33.46 ± 1.37	19.87 ± 3.38	21.92 ± 0.81	22.45 ± 2.44	21.77 ± 0.49
DSL (µm)*	12.96 ± 0.89	12.81 ± 0.45	11.93 ± 1.67	13.43 ± 1.07	6.76 ± 1.65	9.11 ± 0.36	9.12 ± 0.89	8.85 ± 0.71
DAP (µm)*	16.18 ± 0.98	16.17 ± 0.19	15.26 ± 1.55	16.87 ± 0.93	9.69 ± 1.94	11.89 ± 0.35	11.81 ± 1.13	11.69 ± 0.54
VCL(µm/s)*	88.45 ± 2.64^ab^	76.06 ± 1.11^a^	86.53 ± 6.95^ab^	97.15 ± 3.01^b^	47.05 ± 2.60	53.88 ± 1.99	55.98 ± 4.15	54.32 ± 2.60
VSL (µm/s) *	39.83 ± 1.10^ab^	32.51 ± 1.67^a^	37.48 ± 4.43^ab^	42.07 ± 1.92^b^	19.54 ± 1.42	24.11 ± 2.52	25.49 ± 1.37	24.63 ± 1.98
VAP (µm/s) *	47.43 ± 1.22^ab^	39.74 ± 1.43^a^	45.44 ± 4.45^ab^	50.94 ± 1.84^b^	25.77 ± 1.66	30.53 ± 2.38	31.47 ± 1.65	30.86 ± 1.79
BCF(Hz)*	11.77 ± 0.70	10.79 ± 0.13	12.04 ± 1.05	12.71 ± 0.45	6.77 ± 1.02	8.71 ± 0.38	7.86 ± 1.02	7.93 ± 0.61
ALH(µm)*	1.10 ± 0.06	1.02 ± 0.02	1.04 ± 0.07	1.16 ± 0.04	0.64 ± 0.05	0.71 ± 0.02	0.73 ± 0.06	0.69 ± 0.03
STR(%)	0.84 ± 0.00	0.82 ± 0.01	0.82 ± 0.02	0.83 ± 0.01	0.76 ± 0.01	0.78 ± 0.02	0.81 ± 0.01	0.79 ± 0.02
LIN(%)	0.45 ± 0.00	0.43 ± 0.02	0.43 ± 0.02	0.43 ± 0.01	0.40 ± 0.01	0.45 ± 0.03	0.46 ± 0.01	0.46 ± 0.03
WOB(%)	0.53 ± 0.00	0.52 ± 0.01	0.53 ± 0.01	0.52 ± 0.00	0.52 ± 0.00	0.57 ± 0.02	0.56 ± 0.01	0.57 ± 0.02

Values are presented as mean ± SEM. *ALH* anterior lateral head displacement; *BCF* beat cross frequency; *DSL* straight-line distance; *DAP* average path distance; *DCL* curvilinear distance; *LIN* linearity; *STR* straightness; *VSL* straight-line velocity; *VAP* average path velocity; *VCL* curvilinear velocity; *WOB* wobble.* The effect of different doses of vitamin D in normozoospermic bulls is significantly greater than that in asthenozoospermic bulls.^a, b^ Means in a row, with different capital alphabetic letters are significantly different *P* < 0.05. Means without common subscript(s) are not significantly different (*P* > 0.05)

**Table 2:** Effect of supplementation of semen extender with different concentration of vitamin D on frozen–thawed spermatozoa quality (viability and motility) in normozoopsermic and asthenozoopsermic Holstein bulls

Vit D (ng/mL)	Normozoospermic bull				Asthenozoospermic bull			
	**0**	**5**	**10**	**50**	**0**	**5**	**10**	**50**
Total Motility*(%)	71.26 ± 2.83	65.58 ± 2.06	74.95 ± 9.75	82.68 ± 4.75	41.83 ± 12.37	60.60 ± 6.14	52.50 ± 2.45	48.30 ± 3.29
Progressive motility*(%)	53.90 ± 1.14^a^	52.40 ± 1.44^a^	55.63 ± 5.38^a^	65.39 ± 1.56^b^	20.52 ± 2.62^ A^	31.53 ± 0.86^B^	32.29 ± 1.33^B^	31.08 ± 0.14^B^
Immotile*(%)	28.74 ± 2.83	34.42 ± 2.06	25.05 ± 9.75	22.03 ± 0.64	58.17 ± 12.37	39.40 ± 6.14	47.50 ± 2.45	51.70 ± 3.29
Viability (%)	54.33 ± 1.76^a^	52.00 ± 0.58^a^	56.33 ± 3.28^ab^	63.00 ± 2.00^b^	57.00 ± 3.06^B^	45.67 ± 2.96^AB^	37.67 ± 4.26^ A^	41.00 ± 5.00^ A^

Data are expressed as means ± SEM. ^a,b A, B^ Means in a row, with different capital alphabetic letters are significantly different *P* < 0.05

* The effect of different concentration of vitamin D in normozoospermic bulls is significantly higher than asthenozoospermic bulls

### Sperm plasma and acrosomal membrane integrity

**Fig. 1:**
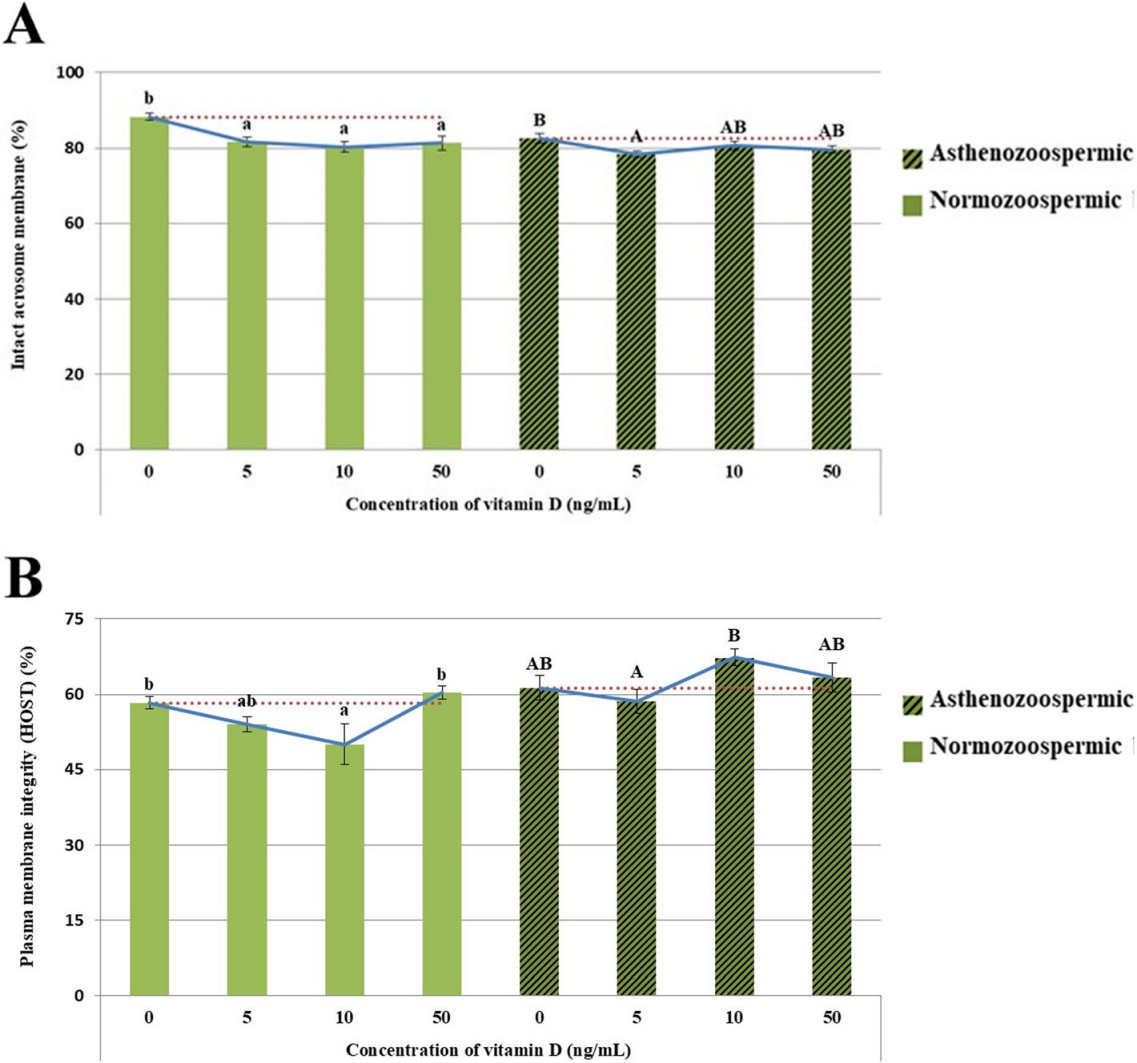
Evaluation Intact acrosome integrity (PMI) of frozen-thawed normozoospermic and asthenozoospermic Holstein bulls spermatozoa supplemented with different concentration of vitamin D. Evaluation Plasma membrane integrity (AI) of frozen-thawed normozoospermic and asthenozoospermic Holstein bulls spermatozoa supplemented with different concentration of vitamin D. *Data are expressed as means ± SEM. a, b, **A**, **B** Different alphabetic letters in each bar indicate statistically significant differences in normozoospermic and asthenozoospermic bulls, respectively. ** There is no significant difference between the effects of different concentrations of vitamin D in asthenozoospermic and normozoospermic bulls, although there is a significant difference between the effects of different concentrations in each group of bulls.

Data of plasma membrane and acrosomal integrity of the two groups are revealed in Fig. 1 (A, B). All doses of vitamin D decreased the rate of acrosome integrity in normozoospermic group (*P* < 0.05). In the asthenozoospermic group, 5 ng/mL vitamin-treated groups could decrease the rate of acrosome integrity compared to control group (Fig. 1 A). A lower rate of plasma membrane integrity was observed in 10 ng/mL of vitamin D- treated group in normozoospermic group compared to control group (Fig. 1B), while this parameter was higher in 10 ng/mL of vitamin D-treated group in asthenozoospermic group compare to 5 ng/ml of vitamin D- treated group.

### Phosphatidylserine externalization assay

**Fig. 2:**
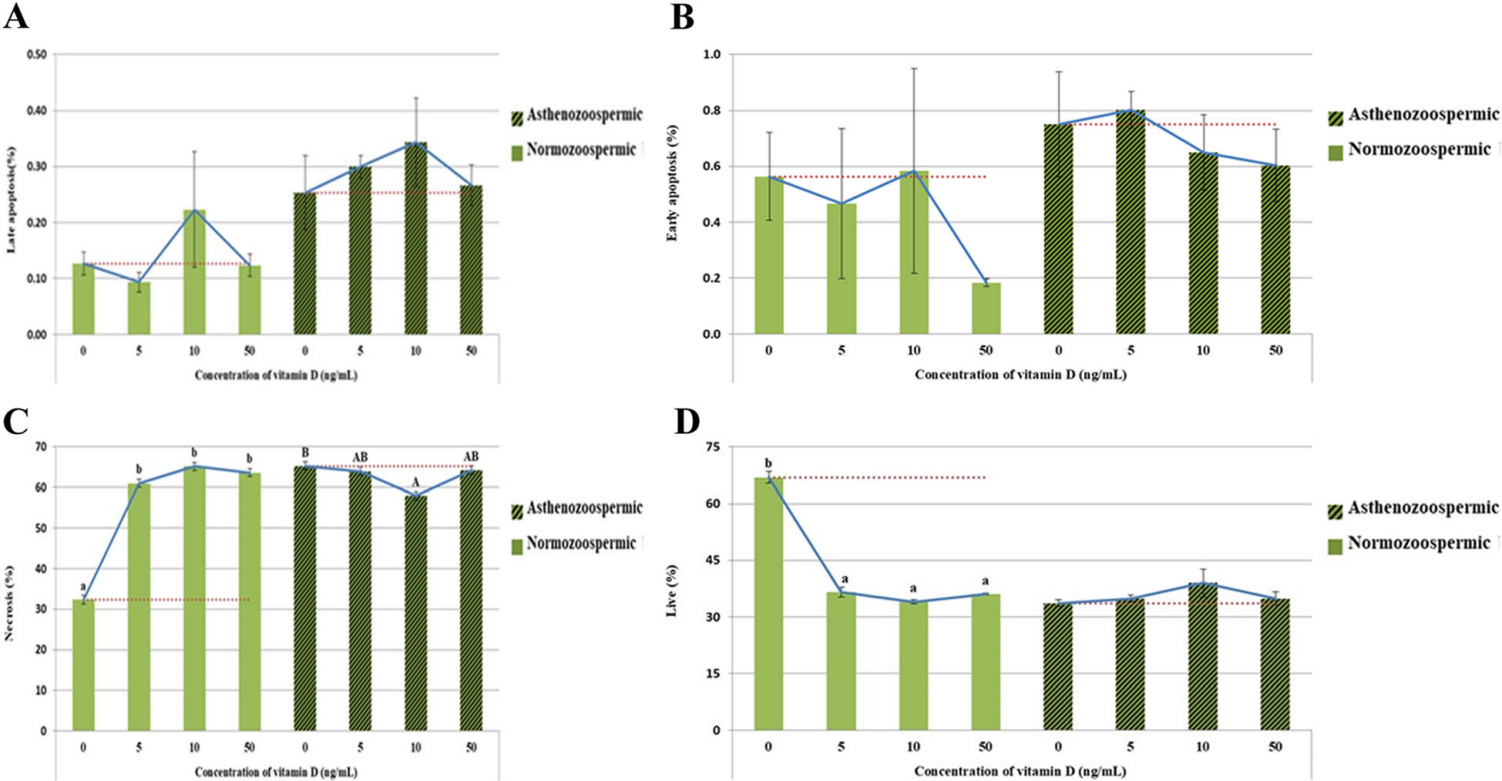
Flow cytometric evaluation of apoptosis statues of frozen-thawed normozoospermic and asthenozoospermic Holstein bulls’ spermatozoa supplemented with different concentration of vitamin D (**A** late apoptosis, **B** necrosis, **C** early apoptosis and **D** Live sperm). ^a,b A,B^ Different alphabetic letters in each bar indicate a significant difference in normozoospermic and asthenozoospermic bulls

Apoptosis statuses are shown in Fig. 2 (A, B, C, and D). Higher percentages of viable spermatozoa were observed in the control group in normozoospermic bulls. (Fig. 2D). The proportions of early-apoptosis (*P* = 0.049) (Fig. 2B) and late-apoptosis (*P* = 0.005) (Fig. 2 A) were significantly higher in the asthenozoospermic group than in the normozoospermic group. There was no statistically significant difference for the normozoospermic group with all doses of vitamin D compared to the control group. There are significant effects of all doses of vitamin D on percentage of necrotic cells in normozoopsermic bulls. (Fig. 2 C). Moreover, there are no significant effects of all doses of vitamin D on percentage of necrotic cells in asthenozoospermic bulls, but the percentage of necrotic sperm was lower in in the 10 ng/mL vitamin D-treated group (*P* < 0.05) compared to control group (Fig. 2 C).

## 3.5. ROS production levels

**Fig. 3:**
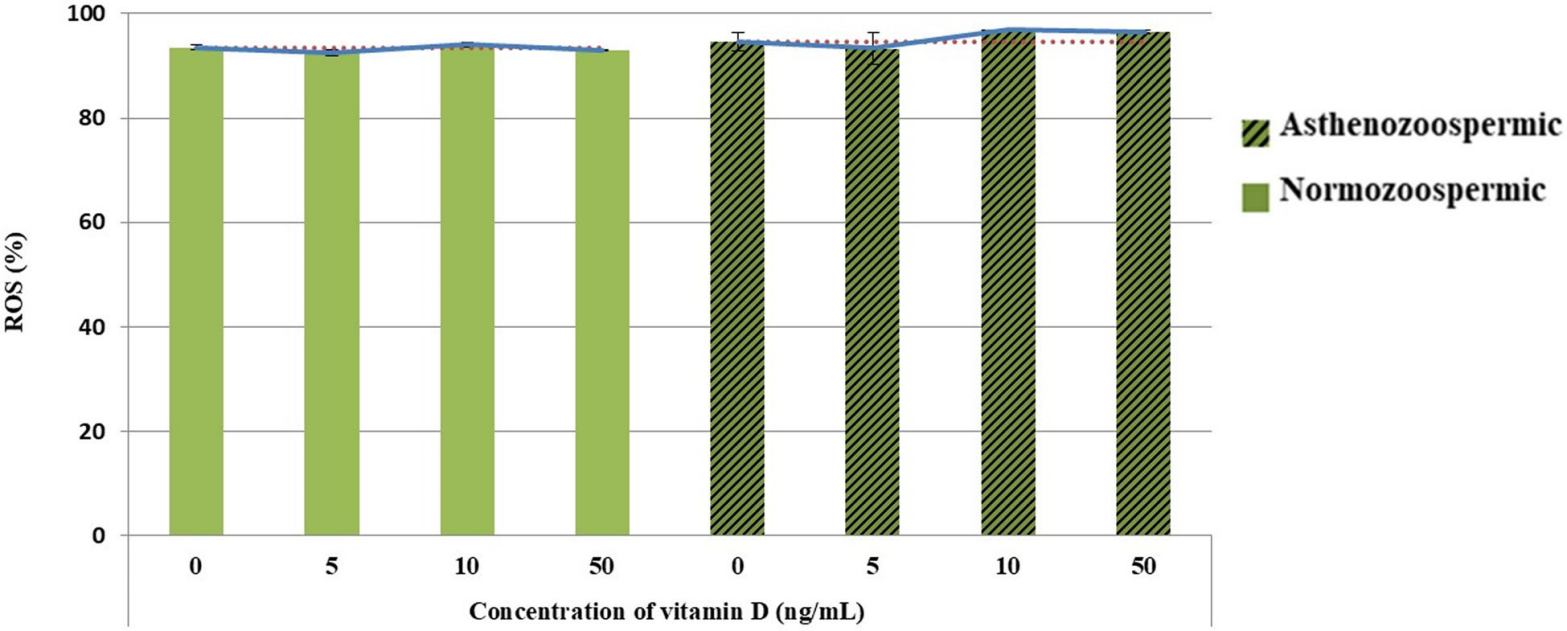
Effect of supplementing semen extender with vitamin D on reactive oxygen species (ROS, %) in post-thawed sperm normozoospermic and asthenozoospermic Holstein bulls. * Mean in columns without different superscripts are not significantly differ (*P* > 0.05)

Results for the percentage of ROS in sperm samples after the freeze-thaw process are presented in Fig. 3. There were no remarkable differences between the normozoospermic and asthenozoospermic groups in terms of the percentage of ROS production.

## Discussion

The current research aimed to determine whether adding vitamin D to the freezing extender could ameliorate the quality of frozen-thawed spermatozoa in normozoospermic and asthenzoospermic bulls, and the protection of spermatozoa by vitamin D against damages during the process of cryopreservation. Sperm cryopreservation, as a useful technique in the assisted reproductive technology (ART), has been used for different purposes, such as fertility preservation for an extended time [26]. However, sperm factors, including motility, morphology, viability, and DNA integrity were usefully decreased following the freeze-thaw process [27]. There is some proof that vitamin D may be involved in regulating sperm quality [28, 29]. However, the role of vitamin D in the semen quality, apoptosis, and ROS in sperms of normozoospermic and asthenozoospermic bulls has not been investigated. The results showed that the supplementation of freezing extender with vitamin D, especially with 50 ng/mL, significantly enhanced progressive motility in both normozoospermic and asthenozoospermic groups.

In this study, adding 50 ng/mL of vitamin D to the semen freezing extender of the normozoospermic bulls improved sperm viability after the freeze-thaw process. In agreement with our results, Moghaddam et al. [18] demonstrated that vitamin D could improve the percentage of human sperm viability after the freeze-thaw process. However, Blomberg-Jensen et al. [30] showed that supplementation with vitamin D and calcium had no effects on semen quality or pregnancy rate in men with vitamin D insufficiency. Previous studies demonstrated that vitamin D increased spermatozoa survival ability by regulating human sperm cholesterol outflows and affecting sperm protein serine and threonine phosphorylation, which effectively enhanced their fertilization ability in the female reproductive tract [28, 11].

Our study revealed the positive effect of a high dose of vitamin D on sperm motility in sperms of bulls with normozoospermia and asthenozoospermia after freezing-thawing process. In agreement with our study, Wadhwa et al. [31] found a positive relationship between vitamin D levels and progressive sperm motility. Many factors play a role in sperm motility regulation, including cyclic adenosine monophosphate (cAMP), which plays a role in modulating mitochondrial function by activating various downstream factors, such as protein kinase A (PKA) [32]. On the other hand, adding vitamin D to the sperm culture medium was reported to increase sperm motility by increasing the levels of calcium in the cytoplasm and mitochondria through an increase in inositol triphosphate (IP3) in spermatozoa [33].

High doses of vitamin D in normozoospermic bulls’ semen had not a positive effect on some kinematic parameters compared to the control, Jueraitetibaike et al. [29] demonstrated that incubation of human sperm with 1,25(OH)2D at a concentration of 0.1 nmol l^− 1^ improved sperm kinetic parameters after 30 min. Under these incubation conditions, the upward migration of spermatozoa enhanced remarkably with increasing adenosine triphosphate (ATP) concentration. Therefore, it can be assumed that these velocity parameters are correlated with sperm fertilization ability and pregnancy rate [34]. However, no significant differences were observed between asthenozoospermic treatment groups in terms of post-thaw sperm motion parameters. These might point to potentially dangerous changes in cryopreserved sperm caused by physical and chemical stress during the freezing-thawing process [35]. Future research should focus on the other concentrations of vitamin D in the dilution medium of asthenozoospermic bull’s semen samples during cryopreservation.

Sperm plasma membrane integrity is essential for assessing sperm function. In the present study, supplementing semen extender with 10 ng/mL of vitamin D enhanced membrane integrity in asthenozoopsrmic bull spermatozoa after the freeze-thaw process. This finding is in agreement with Moghadam et al. [18], who reported that the supplementation of human freezing semen extender with vitamin D improved the percentage of plasma membrane integrity during freezing and thawing by decreasing ROS. Interestingly, these data are in agreement with another study, where adding an antioxidant, such as vitamin E, to cryopreservation extender improved the intact membrane percentage [36]. The mechanism of enhancement of intact membrane plasma may be related to phospholipids, which protect sperm from damage caused by cold shock [37]. This is done by replacing phospholipids in the extender with phospholipids damaged by freezing [38]. Vitamin D acts as a defense system in the cell phospholipid membranes and mitochondrial sheath against oxidative stress [39]. Therefore, a portion of the beneficial effects of vitamin D on the motion parameters of frozen–thaw might be related to the integrity of the plasma membrane.

In this study, different concentration of vitamin D had no effect on the rate of acrosome integrity, and it could not improve acrosome integrity in normozoospermic bull sperm samples. It has been shown that acrosome integrity was important for sperm fertility since an intact acrosome was needed for the incidence of acrosome reactions and sperm penetration into the oocyte [40]. It is well-known that plasma membrane disruption due to ROS results in increased membrane permeability and uncontrolled intracellular ion concentration [41]. Our study illustrated that the supplementation of semen extender with vitamin D could not reduce the ROS production level in normozoospermic and asthenozoospermic bulls. These data are inconsistent with decreased ROS levels by adding vitamin D to the semen extender reported elsewhere [42]. Vitamin D administration in diabetic rats reduced ROS levels by suppressing the NADPH oxidase gene, which is a primary source of ROS and its activation contributes as a positive marker to oxidative stress [43]. Contradictory results in the studies may be due to the duration of the freezing process, freezing method, diluent, and breed of livestock.

In another experiment, the status of apoptosis was measured in the sperm samples of normozoospermic and asthenozoospermic bulls. Our results showed that the proportions of early-apoptosis and late-apoptosis were significantly higher in the asthenozoospermic group than those of the normozoospermic group. The Annexin V indicator of phosphatidylserine translocation can be found in the early stages of membrane damage, which is sufficient for evaluating the responses of sperm cells to stressful conditions, such as cooling and freezing processes [44] and acts as an additional tool to detect the resistance of the male sperm to cryopreservation [45]. In a previous study, apoptosis was increased by DNA fragmentation and phosphatidylserine translocation in frozen sperms, and vitamin D could improve these factors in sperm [18]. Zhang et al. [46] showed that vitamin D had an anti-apoptosis function in some cells. To describe these results, one study showed that vitamin D could react with fatty acid residues in the cell membrane by its hydrophobic parts and could preserve it from decomposition, thereby controlling caspase activation in apoptosis [25].

## Conclusions

The results of the present study revealed that supplementing freezing semen extender with a high dose of vitamin D (50 ng/mL) protected normozoospermic bulls’ sperms from the freezing procedure and improved sperm motility, viability, and plasma membrane integrity. However, the selected doses in this study have no effect on reduced ROS levels in each groups of bull semen. Future research could investigate the molecular pathway and the different protective effects of vitamin D in asthenozoospermic and normozoospermic semen samples during cryopreservation.

The authors would like to thank the Research Council of University of Tabriz for the financial support for this study. We would also like to thank the stuff of the Semen Production Center, Karaj, Iran, for providing the bulls and facilities utilized in this study.

## Data Availability

Data and materials are presented in the materials and methods sections.

## Abbreviations

ANOVA: Analysis of variance
SEM: Standards error of mean
CASA: Computer- assisted sperm analysis
HOST: Hypo –osmotic swelling test
ROS: Reactive oxygen species
DMSO: Dimethyl sulfoxide
ART: Assisted reproductive technology
FITC-PSA: fluorescein-conjugated Pisum sativum agglutinin
